# Recurrent cerebral microbleeds with acute stroke symptoms: A case report

**DOI:** 10.1097/MD.0000000000012480

**Published:** 2018-09-28

**Authors:** Pahn Kyu Choi, Ji Yeon Chung, Seung Jae Lee, Hyun Goo Kang

**Affiliations:** a Department of Neurology, Chosun University School of Medicine and Hospital, Gwang-ju; b Department of Chemistry; c Department of Neurology, Chonbuk National University School of Medicine and Hospital, Jeonju, South Korea.

**Keywords:** cilostazol, microbleeds, MRI, recurrent, stroke

## Abstract

**Rationale:**

Cerebral microbleeds are lesions that appear as round low signal intensity areas with a diameter of 2–5 mm on gradient echo T2-weighted sequence magnetic resonance imaging. Cerebral microblees are hemorrhages found in the brain parenchyma and they are caused by the extravasation of the blood. Although more patients with ischemic stroke are found to have cerebral microbleeds, only a few studies have evaluated other neurologic abnormalities outside of cognitive dysfunction due to cerebral microbleeds.

**Patient Concerns:**

A 73-year-old female patient had only a lacunar infarction with the development of a new microbleed whenever a new neurologic symptom occurred, without the occurrence of acute ischemic stroke.

**Diagnoses:**

A 73-year-old female patient diagnosed symptomatic cerebral microbleeds.

**Interventions:**

Brain magnetic resonance imaging was taken within a few hours of the occurrence of a new symptom and we confirmed increased cerebral microbleeds in the ventral-posterolateral area of the thalamus, consistent with the symptoms.

**Outcomes:**

This case study is meaningful because it proves that repeated occurrences of cerebral microbleeds in a specific area can induce acute ischemic stroke-like symptoms.

**Lessons:**

Cerebral microbleeds have been considered to be asymptomatic lesions thus far. However, recent studies have reported the association of cerebral microbleeds with neurological symptoms including cognitive dysfunction. This study confirmed the presence of newly formed cerebral microbleeds through imaging follow-ups whenever a symptom occurred.

## 1 Introduction

Cerebral microbleeds (CMBs) are lesions that appear as round low signal intensity areas with a diameter of 2 to 5 mm on gradient echo image (GRE) T2-weighted imaging sequence magnetic resonance imaging (MRI).^[1]^ CMBs are hemorrhages found in the brain parenchyma and they are caused by the extravasation of the blood vessel. It is believed that they exhibit a low signal intensity on GRE because of the paramagnetic effects of blood breakdown products including hemosiderin, deoxyhemoglobin, and ferritin.^[2]^ Although more patients with ischemic stroke are found to have CMBs,^[3]^ only a few studies have evaluated other neurologic abnormalities outside of cognitive dysfunction due to CMBs.^[4]^ The authors present the case of a patient who had only a lacunar infarction with the creation of a new CMB whenever a new neurologic symptom occurred without the occurrence of acute ischemic stroke. Therefore, we report this case with a review of the previous literature.

## 2 Case report

**Figure 1:**
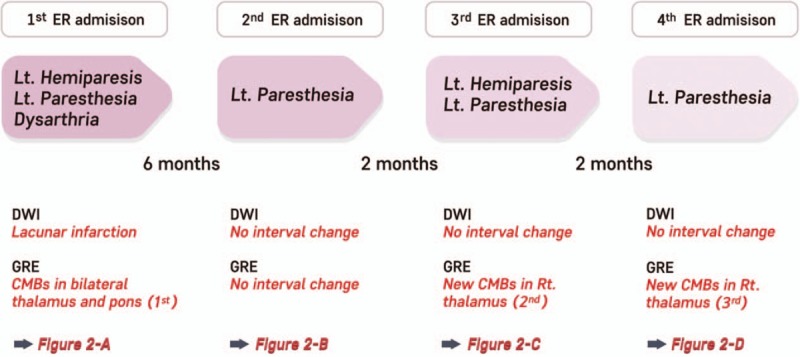
Timeline of the patient. DWI = diffusion-weighted image, ER = emergency room, GRE = gradient echo image, Lt = left.

**Figure 2:**
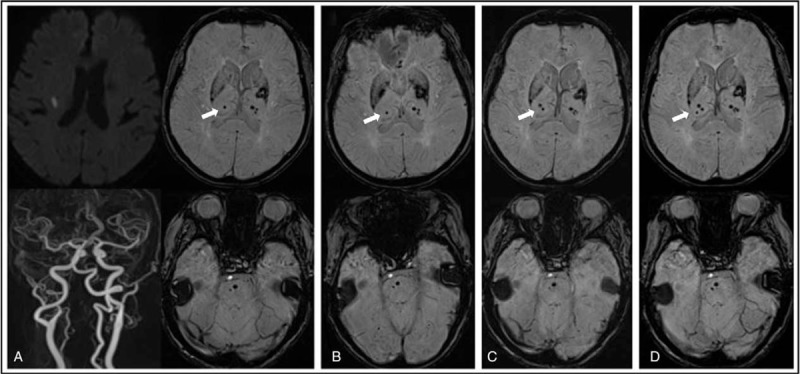
Brain images of the patient. (A) Brain MRI with acute ischemic stroke. Brain DWIs show right lenticulostriatal artery territorial infarction. Multiple microbleeds are seen in the bilateral deep gray matter (arrow) and pons on GREs. (B) The newly taken DWI and GRE brain MRI do not appear different from previous images (arrow). (C) Eight months after the previous ischemic stroke, a new microbleed was observed (arrow) in addition to previous microbleeds in the right thalamus on GRE, with the patient presenting with left hemiparesis and paresthesia. (D) Two months after the previous microbleed, a new microbleed was observed (arrow) in addition to previous microbleeds in the right thalamus on GRE, with the patient again presenting with left paresthesia. DWI = diffusion-weighted image, GRE = gradient echo image, MRI = magnetic resonance imaging.

A 73-year-old female patient visited the hospital due to left-sided hemiparesis. She did not have a family history of stroke and had been taking antihypertensive medication for the past 10 years and angina medication for the past 6 years. She had a chronic headache for past few years. She had frequent headache with nausea or vomiting on 15 days per month. A neurological examination was conducted, and left hemiparesis, paresthesia, and dysarthria were found (Fig. 1). The muscle power of the left upper and lower limbs was Medical Research Council (MRC) grade III and the brain diffusion-weighted MRI (diffusion weighted image [DWI]) showed a right lenticulostriate artery territorial infarction. Obstruction and stenosis of the main vessel were not observed. Multiple CMBs were found in the bilateral deep gray matter and pons on GRE MRI (Fig. 2A). Transthoracic echocardiography was normal. Cilostazol 50 mg twice daily was administered for secondary prevention of stroke in consideration of the multiple CMBs. The muscle power of the patient's left upper and lower limbs improved to MRC grade IV on the 7th day of hospitalization so she was discharged. Outpatient follow-up examination found that the muscle power of patient's left upper and lower limbs improved to MRC grade V 1 month after discharge. However, her hypertension was not controlled. Therefore, the dose of existing hypertension medication was increased and the follow-up examination found that her blood pressure was well controlled afterward.

The patient presented with numbness in the left upper limb 6 months after discharge, and DWI and GRE brain MRI were performed. The newly taken DWI and GRE brain MRI were not different from previous images (Fig. 2B). Eight months after discharge, the patient experienced acute left hemiparesis and paresthesia with headache and she visited the emergency room within 1 hour of its onset. Neurological examination revealed that the muscle strength of the left upper and lower limbs was decreased to MRC grade IV. The blood pressure of the patient was 200/110 mm Hg when she visited the emergency room and electrocardiography did not show any abnormal findings except sinus bradycardia. The blood test was normal. The recurrence of cerebral infarction was suspected so brain MRI and DWI were performed but an acute infarction was not found. However, a new microbleed was observed in addition to previous CMBs in the right thalamus on the GRE sequence (Fig. 2C). The muscle power of the patient's left upper and lower limbs improved to MRC grade V from the 2nd day after admission and the patient was discharged 3 days later. After admission, the patient's blood pressure was not well controlled and she complained of headache. Therefore, the dose of previous hypertension medication was adjusted again, and the blood pressure was well controlled afterward. Outpatient follow-up found that the left paresthesia improved to normal, as well improvements with headache.

Two months after the occurrence of the new CMBs, the patient experience left-sided paresthesia and visited the emergency room within 2 hours of its occurrence. Neurological examination was performed on presentation and found that the muscle power of the left upper and lower limbs was normal (MRC grade V) but paresthesia, which was previously improved, and headache occurred again. Her blood pressure was 210/110 mm Hg on admission and electrocardiography and blood test results were normal. Laboratory studies and abdominal ultrasonography for evaluation of secondary hypertension were normal. Brain MRI was obtained again to confirm the recurrence of cerebral infarction. No new lesions were seen on DWI. However, it was confirmed that another new microbleed had occurred, in addition to the 2 existing CMBs in the right thalamus on GRE MRI (Fig. 2D). After admission, her blood pressure was too high and the dose of hypertension medication had to be adjusted again. The blood pressure was well maintained afterward. The patient's paresthesia and headache improved from the next day, and the patient was discharged 5 days later. The left-sided paresthesia began to improve from the 2nd week after discharge and she has been treated as an outpatient without recurrence of neurological symptoms. Patient was followed up for 12 months at the outpatient clinic. Blood pressure was well controlled and there was no abnormal neurological symptoms.

## 3 Discussion

The lesions found in the GRE images of this case were homogenous, round, and had low signal intensity, consistent with the diagnosis of CMBs. Calcification and iron deposits appear as small low signal intensity foci on T2-weighted MRI and illustrate mainly symmetric low signal in subcortical areas and the basal ganglia. Moreover, the flow voids of pial blood vessels exhibit a linear shape. They are different from CMBs, which mainly appear as a blind-ended round or ovoid structure. Low signal intensity lesions were found in the same area in the repeated GRE brain MRIs. Therefore, the possibility of a partial volume artifact could be excluded. Cavernous malformation showing heterogeneous low signal intensity could be excluded as well.^[5]^

CMBs are known as silent lesions^[6]^ but recent studies have reported that CMBs are related to cognitive dysfunction.^[4]^ No large-scale study has shown that CMBs cause other neurological abnormalities except cognitive dysfunction. Some studies have reported that stroke-like neurologic abnormalities were induced by CMBs but the temporal causal relationship of CMBs and neurological abnormalities was not clear in these cases (Table 1).^[7,8]^ Although Teo et al reported a case of CMBs without acute cerebral infarction in a location consistent with the symptom,^[8]^ it was not possible to know if the CMBs existed before the onset of the symptom because there was no brain MRI before the occurrence of the new symptom. Consequently, previous studies could not prove whether the symptom was caused by CMBs. On the other hand, in this case, we obtained brain MRI within a couple of hours of the occurrence of a new symptom and confirmed the increased CMBs in the ventral-posterolateral area of the thalamus, consistent with the symptoms. Moreover, the lesion correlated with the new symptoms. These are important findings, particularly the new symptoms could be the common fluctuation of residual symptoms due to the sequelae of existing cerebral ischemic stroke. However, it is noteworthy that new CMBs were observed in the area associated with the symptoms.

**Table 1:**
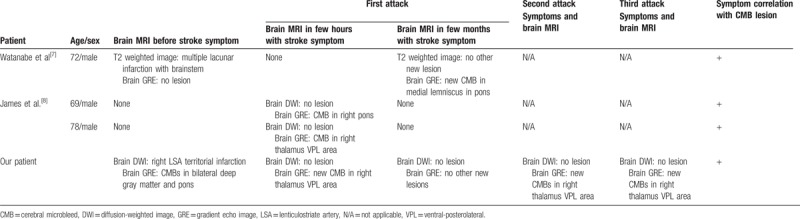
Brain magnetic resonance image of acute stroke-like symptoms with CMBs.

CMBs are hemorrhages in the brain parenchyma where hemosiderin deposits locally around the small vessel. Hypertensive arteriopathy and cerebral amyloid angiopathy are known as the main causal mechanisms of CMBs. Lobar CMBs generally occur in cerebral amyloid angiopathy and CMBs in the deep gray matter are associated with hypertensive arteriopathy.^[9]^ CMBs are also known as indicators of small vessel disease.^[10]^ In particular, CMBs are highly associated with small vessel disease when they are located in the deep gray matter or infratentorial area.^[11]^ The occurrence mechanisms of lacunar infarction and white matter change are similar to those of CMBs,^[12]^ because they are also caused by the extravasation of the blood vessel and blood components due to damage to the arterial endothelium.^[13]^ Moreover, hypertension is known as an important causal factor of lacunar infarction and white matter change, while CMBs can also be caused by hypertension.^[9,13]^ The causal mechanisms of CMBs are similar to those of small vessel disease or lacunar infarction.

It is believed that CMBs, which typically follow the path of the corticospinal tract, easily causing symptoms, even with a small lesion, or are located in areas such as the thalamus, would cause symptoms similar to an ischemic stroke. The patient in this case had CMBs in the thalamus as well as lacunar infarction. Furthermore, she had uncontrolled hypertension and experienced acute stroke symptoms and headache several times when her blood pressure was not controlled well. Whenever she experienced a symptom, new CMB lesions were observed in the thalamus. The dosage of antiplatelet was maintained continuously and the dosage of antihypertensive agent was increased during each admission. New neurological symptoms and headache were not observed in follow-up after her hypertension was controlled. Therefore, the authors suspect that uncommon symptomatic CMBs occurred due to hypertensive arteriopathy because her hypertension was not controlled well. Moreover, all neurological symptoms owing to the CMBs improved within a few days in this case. These results suggest that neurological symptoms due to CMBs could have improved rapidly as the extravasation of the small vessel hemorrhage improved, unlike the symptoms of ischemic stroke, which mostly remain because of the sequelae of cell death.

Cilostazol is known to act as an antiplatelet agent and enhance endothelial function.^[14]^ Our patient had taken cilostazol 100 mg twice a day after acute ischemic stroke throughout the outpatient follow-up. Therefore, endothelial cell stability was improved, and this may be helpful for the rapid recovery of neurological symptoms due to CMBs. However, it is impossible to generalize that CMBs cause neurological symptoms only based on the results of this case study, because it is a single case and not a large-scale prospective study. Therefore, it will be necessary to have a large-scale study of the relationship between the blood pressure control of patients with lacunar infarction and the occurrence of symptomatic CMBs in the future.

CMBs have been considered to be asymptomatic lesions thus far. However, recent studies have reported the association of CMBs with neurological symptoms including cognitive dysfunction. However, as far as the authors are aware, no study has confirmed that the number of new CMBs lesions increased with the occurrence of repeated acute ischemic stroke-like symptoms based on imaging evidence. This case study is meaningful because it proves that repeated occurrences of CMBs in a specific area can induce acute ischemic stroke-like symptoms and this study confirmed the presence of newly formed CMBs through imaging follow-ups whenever a symptom occurred. The results of this study suggest that a large-scale prospective study will be needed to determine the symptom occurrence according to the location of CMBs.

## Author contributions

**Conceptualization:** Pahn Kyu Choi, Ji Yeon Chung, Hyun Goo Kang.

**Data curation:** Hyun Goo Kang.

**Formal analysis:** Hyun Goo Kang.

**Methodology:** Pahn Kyu Choi, Ji Yeon Chung, Seung Jae Lee, Hyun Goo Kang.

**Resources:** Pahn Kyu Choi, Ji Yeon Chung.

**Supervision:** Seung Jae Lee.

**Validation:** Ji Yeon Chung, Hyun Goo Kang.

**Writing – original draft:** Pahn Kyu Choi.

**Writing – review & editing:** Ji Yeon Chung, Seung Jae Lee, Hyun Goo Kang.

Hyun Goo Kang orcid: 0000-0001-5443-3635
